# Extracellular Vesicles From Stored Red Blood Cells Convey Heme and Induce Spic Expression on Human Monocytes

**DOI:** 10.3389/fimmu.2022.833286

**Published:** 2022-05-18

**Authors:** Carolinne Souza Amorim, João Alfredo Moraes, Ingrid de Jesus Magdalena, Sheila Gutiérrez López, Ana Carolina Dudenhoeffer Carneiro, Isabelle Karine da Costa Nunes, Luciana Pizzatti, Vinícius Figueiredo Sardela, Francisco Radler Aquino Neto, Luciana Cristina Mirotti, Henrique Marcelo Gualberto Pereira, Mariana Renovato-Martins

**Affiliations:** ^1^ Laboratório Brasileiro de Controle de Dopagem-Laboratório de Apoio ao Desenvolvimento Tecnológico (LBCD-LADETEC), Instituto de Química, Universidade Federal do Rio de Janeiro (UFRJ), Rio de Janeiro, Brazil; ^2^ Laboratório de Biologia Redox, Programa de Pesquisa em Farmacologia e Inflamação, Instituto de Ciências Biomédicas, Universidade Federal do Rio de Janeiro, Rio de Janeiro, Brazil; ^3^ Laboratório de Biologia Molecular e Proteômica do Sangue-Laboratório de Apoio ao Desenvolvimento Tecnológico (LABMOPS-LADETEC), Instituto de Química, Universidade Federal do Rio de Janeiro (UFRJ), Rio de Janeiro, Brazil; ^4^ Laboratório de Inflamação e Metabolismo, Departamento de Biologia Celular e Molecular, Instituto de Biologia, Universidade Federal Fluminense, Niterói, Brazil

**Keywords:** extracellular vesicles, monocytes, Spic, heme, red blood cells, autologous blood transfusion

## Introduction

During cold storage, the conditions to which red blood cells (RBC) are exposed, such as temperature and composition of the medium, are different from *in vivo* conditions culminating in non-physiological aging (1). As a result, RBC can undergo irreversible morphological changes, hemolysis, or even degradation with a concomitant increase in extracellular free-iron, heme, hemoglobin, and RBC-derived Extracellular Vesicles (EVs), the so-called storage lesions. Detoxification systems become overwhelmed when large amounts of free heme or hemeproteins accumulate (2). In this context, macrophages have a pivotal role as the primary cells responsible for the uptake and disposal of excess heme resulting from hemolysis, the recognition of RBC-derived EVs, and the clearance of damaged RBCs from circulation (3, 4). Heme is metabolized inside monocytes/macrophages by the sequential activity of the inducible enzyme heme oxygenase (HO)-1, which levels are almost undetectable under basal state (5–8).

Monocytes/macrophages are widely distributed and are versatile cells of the innate immune system characterized by high phagocytic activity and roles in tissue homeostasis, control of immune responses, and wound repair (9). For example, macrophages in the spleen, liver, and bone marrow prevent heme-mediated toxicity by phagocyting old erythrocytes, take up heme and hemoglobin released from erythrocytes, and are responsible for iron recycling (7). Furthermore, elevated levels of heme in the red pulp area of the spleen induce the expression of the transcription factor Spic, which is required to develop monocytes into iron recycling macrophages (8). Apart from that, damage-associated molecular patterns (DAMPs) triggered responses induce Spic in monocytes and macrophages (10). For example, increased levels of Spic have been reported in monocytes undergoing M2-like differentiation (anti-inflammatory profile), suggesting its role in macrophage specification and activation (11). Corroborating that, Spic restrains inflammatory response in macrophages and is induced *via* toll-like receptor (TLR) signaling in monocytes (10, 12). Alam et al. have demonstrated that Spic has a role in the transcriptional circuit within activated macrophages by sensing its inflammatory milieu (10). Under inflammatory conditions, tissue macrophages are constantly replenished by circulating monocytes (13); this differentiation depends on the local microenvironment to formulate a context-appropriate response (14). For example, tissue macrophages known as wound-healing macrophages are “M2-polarized”, representing one extreme phenotype from a broad spectrum of macrophage polarization, while proinflammatory “M1-polarized” induce and support immune responses and represent the other extreme (15, 16).

EVs, which include microparticles (0.1-1µm) and exosomes (30-100 nm), carry diverse cargoes, including proteins, lipids, and RNA species that can be exchanged between cells, playing a role in cell-to-cell communication (17). Camus et al. have demonstrated that in sickle cell disease (SCD), microparticles from RBC deliver toxic heme to endothelial cells, increasing the production of reactive oxygen species, adhesion molecules, and apoptosis (18). Even though the clinical relevance of EVs released by red blood cells (RBC-EVs) to transfusion safety is still unknown, they could potentially prime the recipient’s immune system of the recipient, so further investigations are needed.

Apart from clinical indications for blood transfusion, cheating athletes perform blood transfusions to increase their performance, being recognized as an illicit practice under the name of blood doping, which is prohibited by the World Anti-Doping Agency (WADA) (19). In autologous blood transfusion (ABT), RBC are removed from athlete’s circulation, stored (refrigerated or frozen) until needed, and then reinfused Since the transfusion of one or two bags of blood might prove difficult to detect (20, 21), there is currently no method capable of effectively detecting this practice. Thus, developing novel and sensitive methods for doping control purposes is demanded.

In the present study, through an *ex vivo* blood transfusion protocol, we aimed to investigate the immunomodulatory effects of EVs derived from previously-stored RBC under monocytes and highlight a possible marker for detecting ABT in monocytes. The results have shown that stored RBC release increased amounts of heme-enriched EVs, which induce Spic expression in monocytes. Furthermore, we call attention to Spic detection as a feasible doping detection approach.

## Material and Methods

### Research Participants

For this study, thirteen (13) healthy volunteers (4 male, 9 female) aged 20 - 55 years, with a body mass index (BMI) between 18 and 30, were recruited from the Laboratório Brasileiro de Controle de Dopagem (LBCD) or Laboratório de Biologia Redox (LabiO-RedOx) from Federal University of Rio de Janeiro. Ethics committee approval: CAAE 36880914.0.0000.5259.

### Red Blood Cells (RBCs)

Blood was collected from healthy volunteers in EDTA anticoagulant BD vacutainer tubes. After this, tubes were maintained for approximately 20 minutes on a roller-type shaker (Kasvi, São José dos Pinhais, Brazil) for complete homogenization. The whole blood (containing plasma) was stored for 21 days at 4°C in an acid-citrate-dextrose (ACD) preserving solution in a ratio of 1:7 (ACD: whole blood). Blood from the same donor was collected on the last day of storage, subjected to ACD preserving solution 1:7 (ACD: whole blood), and analyzed fresh (day 0). Fresh and refrigerated blood were separately allocated into falcon tubes and centrifuged for 10 minutes at 580 g at room temperature. After centrifugation, the plasma was stored (at -80°C) for subsequent quantification of heme, hemoglobin, and EVs. RBCs were counted in a hematological analyzer (XT2000i™, Sysmex, Kobe, Japan).

### Monocyte Preparation

In the present study, we are investigating monocytes activation, to this fasting venous blood samples (10 mL) were collected from all subjects in BD vacutainer tubes and carefully layered onto Ficoll/Hysto-paque gradients (GE Healthcare, Chicago, USA), followed by centrifugation at 770 g for 30 minutes, at room temperature. Isolated peripheral blood mononuclear cells (PBMCs) were resuspended in RPMI 1640; 10% fetal bovine serum (FBS), seeded at a density of 4 x 10^5^ cells/well in 24-well plates and allowed to adhere for 1 hour at 37°C with 5% CO_2._ After that, non-adherent cells were removed by aspiration, and monocytes were left adhered.

### Isolation and Quantification of Extracellular Vesicles

EVs were isolated from the plasma of fresh blood samples or blood previously stored at 4°C for 21 days. First, the samples were centrifuged (2,000 g for 10 minutes at 4°C) for the removal of cell debris (Eppendorf 5415R, Hamburg, Germany). Thereafter, the obtained supernatant was centrifuged again (20,000 g, for 70 minutes, at 4°C) (Beckman, GS-15R Centrifuge). A control sample containing 1 μm microspheres (Life Technologies, Carlsbad, CA, USA) was used to accurately define the FSC/SSC profile containing the MPs (<1 μm events) with adequate accuracy on the flow cytometer. Samples were labeled with the CD235a-FITC antibody (anti-glycophorin) at 1:100 concentration (clone 11E4B-7-6, IM2212U, Beckman Coulter, France) and 1 μm microspheres (Invitrogen, Waltham, USA) were labeled at 1: 100 concentrations with the FITC Mouse IgG1 isotypic control (clone MOPC 21, BD Biosciences, New Jersey, USA). A total of 100 μL/sample was acquired on the Accuri c6™ cytometer (BD Biosciences, CA, USA). RBC-EVs were also resuspended in sterile PBS and stored at -20°C until use.

### Hemoglobin Oxidation

Hemoglobin (H-2625, Sigma Aldrich, St Louis, Missouri, USA) was oxidized as previously described (22). Briefly, Hb at a concentration of 50 µM heme group was oxidized with H_2_O_2_ (125 µM) (Sigma Aldrich, St Louis, Missouri, USA) for 10 minutes at room temperature (RT).

### *Ex Vivo* Stimulation of ABT

Fresh red blood samples (recipients) were mixed with 21-day-stored blood samples (reinfusion) as follows: 80% of fresh blood with 20% of stored blood (Mix 1) or 90% of fresh blood with 10% of stored blood (Mix 2). These proportions were chosen to simulate a reinfusion of one or two blood bags, respectively.

### Co-Culture of Monocytes (Mo) With RBCs

**Figure 1:**
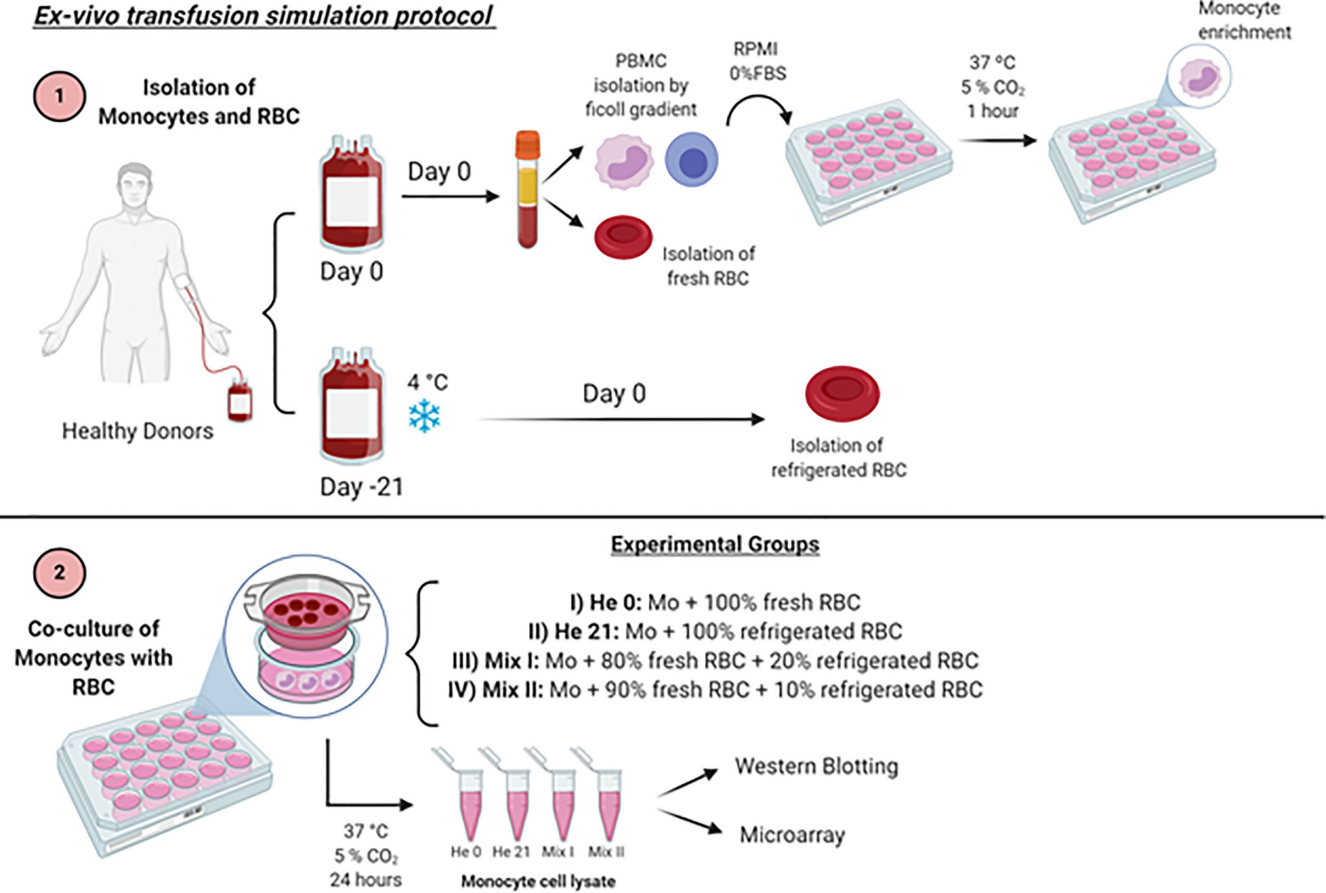
Schematic representation of ABT *ex vivo* protocol. Created with BioRender.com.

After adherence of the monocytes to the plate, they were co-cultured with RBCs in a ratio of 10^3^ RBCs/monocyte (Mo) through a 0.4 μm insert (3422, Corning COSTAR, NY, USA) for 24 hours, at 37°C, under four different experimental conditions (**Figure 1**)

i. RBC 0: Mo co-cultured with 100% fresh RBCs (RBC 0)
ii. RBC 21: Mo co-cultured with 100% 21-day-stored RBCs (RBC 21)
iii. Mix I: Mo co-cultured with 80% fresh RBCs + 20% refrigerated RBCs
iv. Mix II: Mo co-cultured with 90% fresh RBCs + 10% refrigerated RBCs

### Co-Culture of Monocytes With Extracellular Vesicles

After adherence of the monocytes to the plate, they were incubated with EVs for 24 hours at 37°C under different experimental conditions.

i. Untreated Mo (incubated with PBS 20% v/v)
ii. Mo stimulated with EVs from fresh RBC (RBC 0-EVs; 20% v/v)
iii. Mo stimulated with EVs from 21-day-stored RBC (RBC 0-EVs; 20% v/v)

### RNA Extraction, Reverse Transcription, and cDNA Preamplification

Total RNA was isolated from the co-cultured monocytes subsets using the RNeasy Mini Kit (Qiagen, Hilden, Germany) according to the manufacturer’s instructions, including DNAse I treatment. RNA quality and quantity were analyzed on a Qubit 4 fluorimeter (ThermoFisher, Waltham, USA). Samples with A260/A280 ratios between 1.8 and 2.2 were considered suitable for qPCR. RNA was retrotranscribed into cDNA using the High-Capacity cDNA reverse transcription kit (Applied Biosystem, ThermoFisher, Waltham, USA) according to the manufacturer’s instructions.

### Real-Time Polymerase Chain Reaction (RT-PCR)

Complementary DNA samples were amplified using Made-to-Order Low-Density Array System according to the instructions of the manufacturers (Applied Biosystems, ThermoFisher, Waltham, USA). The arrays were carried out on the QuantStudio^®^ 12K Flex-Real-Time PCR System (Applied Biosystems, ThermoFisher, Waltham, USA). Taqman Low-Density Array was used to screen for changes in gene expression of cytokines, chemokines, surface receptors, heme, and Fe metabolism, and others. TaqMan LDAs (Applied Biosystems, ThermoFisher, Waltham, USA) are created on a 384-well microfluidic card, which is arrayed with TaqMan gene expression assays. The experimental design involves an array of 22 essential genes and 18S and actin as endogenous controls as follows. ACTB (Hs99999903_m1), 18S (Hs99999901_s1), TFRC (Hs99999911_m1), FTL (Hs00830226_gH), HPRT1 (Hs99999909_m1), ANXA5 (Hs00154054_m1), HMOX1 (Hs01110250_m1), OXSR1 (Hs00178247_m1), NOS2 (Hs01075529_m1), CCL3 (Hs00234142_m1), HIF1A (Hs00936371_m1), CSF1 (Hs00174164_m1), TNF (Hs01113624_g1), MIF (Hs00236988_g1), IL6 (Hs00985639_m1), IL4 (Hs00174122_m1), CD14 (Hs02621496_s1), CXC3R1 (Hs01022583_s1), CCR2 (Hs01560352_m1), CCR5 (Hs00152917_m1), MRC1 (Hs00267207_m1), CCR3 (Hs00266213_s1), MRC1 (Hs00267207_m1), NEF2L2 (Hs00975961_g1). The assays were predesigned and validated by Applied Biosystems. The relative quantification of each target gene mRNA compared with actin and 18S was calculated as the average 2−ΔCt where ΔCt =Ct−Ct _(mean: actin and 18S)._ The threshold cycle Ct was automatically given by the software Thermocloud dashboard online tool (available at https://apps.thermofisher.com).

### Obtention of Cell Extracts

Monocytes were incubated with RBC or EVs for 24 hours. In specific assays, monocytes were pretreated with TAK-242 (1µM) (Cayman Chemical Company, Ann Arbor, USA) or DMSO (0.001%) for 15 min and then incubated with EVs for 24 hours; or were incubated with RBC-0 EVs, and RBC-21 EVs pretreated with hemopexin (1 µM for 1 hour; R&D Systems, Minneapolis, USA); or incubated with oxidized hemoglobin (metHb) at a concentration equivalent to 30 µM of heme. Thereafter, the wells were washed thoroughly with PBS (Lonza, Walkersville, USA) previously heated at 37°C. Subsequently, PBS was removed, and cells were lysed in RIPA lysis buffer (Sigma-Aldrich, St. Louis, USA) with 1:10 protease inhibitor (P2714-1BTL, Sigma-Aldrich, St. Louis, USA). The lysed samples were placed in 500 μL conical bottom tubes and stored in a freezer at -30°C.

### Electrophoresis and Immunoblotting

The total protein content of the cell extracts was determined by the BCA method (Abcam, ab102536, Cambridge, USA). Cell lysates (10-20 μg of protein) were fractionated by polyacrylamide 12 or 15% (SDS-PAGE) gels (1 hour at 150V and 50mA, PowerPac™ HC, Bio-Rad, Singapore). The proteins were transferred to polyvinylidene difluoride membranes (Immun-Blot™ PVDF, Bio-rad, USA) in Trans-Blot^®^ Turbo™ equipment (15V, 328mA and 30 minutes; Transfer System, BioRad, Singapore). Then, membranes were blocked with 5% BSA (Sigma-Aldrich, St. Louis, USA) in 0.1% T-TBS and incubated overnight at 4°C in the presence of the following primary antibodies: anti-Spic (rabbit - 1: 1000) (Abcam, Cambridge, USA), anti-HO1 (rabbit-1: 1000) (Abcam, Cambridge, USA), anti-IκB (rabbit – 1:1000) (Santa Cruz Biotechnology, Texas, USA), anti-Ferritin (rabbit- 1: 250) (Santa Cruz Biotechnology, Texas, USA) or anti-β-actin (rabbit - 1: 1000) (Abcam, Cambridge, USA). The membranes were incubated for at least one hour with a specific peroxidase-conjugated anti-rabbit secondary antibody (goat - 1: 10000) (Abcam, Cambridge, USA). Immunoreactive proteins were visualized by detection of their chemiluminescence through enhanced chemiluminescence (ECL; SuperSignal™ West Femto Maximum Sensitivity Substrate, ThermoFisher Scientific, Rockford, USA) solution. Membranes were developed and photographed using ImageQuant™ LAS500 (GE Healthcare, Buckinghamshire, England), and densitometry was quantified using ImageJ software (NIH, Bethesda, USA).

### Quantification of Free Heme, Hemoglobin, and Oxidized Hemoglobin

Fresh and 21-day-stored blood samples were centrifuged for 10 minutes x 500 g to separate the plasma. Subsequently, plasma and purified EVs were collected and analyzed at 398 nm (18) and 540 nm for heme and hemoglobin (Hb) concentrations, respectively, using the Envision™ equipment (PerkinElmer, Waltham, MA USA). The results were obtained in absorbance and calculated in μM. The determination of oxidized hemoglobin (metHb) within EVs was calculated as previously described (22, 23). Briefly, the concentration of metHb were monitored using Flexstation™ multilabel plate reader (Molecular Devices, San Jose, CA, USA), and Hb redox forms were measured at 541, 576 and 630 nm by the following equation: [metHb] = 185.77 x OD_541_+ 171.88 X OD_576_ +387.58 X OD_630._ The results were obtained in absorbance and calculated in mM.

### Monocyte’s Uptake EV Assay

Monocytes (1.2 x 10^6^ cells/mL) were plated in 24-well plates and were incubated with EVs for 30 minutes (20% v/v). After treatment, the cells were lysed in radioimmunoprecipitation assay buffer containing SIGMAFAST™ Protease Inhibitor Cocktail Tablet, EDTA-Free (Sigma-Aldrich, St. Louis, USA) on ice for 20 minutes. Heme was measured at 398 nm using the Envision™ equipment (PerkinElmer, Waltham, MA, USA). The results were obtained in absorbance and calculated in μM.

### Reactive Oxygen Species (ROS) Production Analysis

Monocytes (2.5 x 10^5^cells/mL) were suspended in Hank’s balanced salt solution (HBSS) without Phenol Red (Gibco, ThermoFisher, Waltham, USA0. To detect the intracellular ROS production, cells were loaded with a 5 µM CellROX probe (ThermoFisher, Waltham, USA) for 30 minutes prior to treatment. The medium with non-internalized probe was removed and the cells were pretreated with or without TAK-242 (1 µM; Cayman Chemical Company, Ann Arbor, MI, USA) and then incubated with RBC 0-EVs or RBC 21-EVs for 15 minutes at 37°C in a 5% CO_2_ atmosphere During treatment, probe oxidation was monitored using a Flexstation™ Multi-label Plate Reader (ThermoFisher, Waltham, USA) at excitation and emission wavelengths of 640 and 665 nm, respectively.

### NF-kB Activation

Monocytes (2x10^4^ cells/well) were seeded in 96-well plates in RPMI-1640 containing 10% FBS were transfected with the NF-kB-responsive luciferase reporter construct (NF-kB pMetLuc 2) and control plasmid (pMetLuc 2) (Clontech, Mountain View, CA, USA) in RPMI-1640 containing 10% FBS and incubated for 24 h. Cells were pretreated with or without TAK-242 (1 µM; Cayman Chemical Company, Ann Arbor, MI, USA) and then incubated with RBC 0-EVs or RBC 21-EVs for 24 h at 37°C in a 5% CO_2_ atmosphere. The medium containing luciferase was collected for each treatment and was incubated with luciferin. Luminescence emitted from the luciferin cleavage was monitored using the Flexstation™ multilabel plate reader (Molecular Devices, San Jose, CA, USA).

### Heme Analysis by Metabolomics

#### Sample Extraction

Metabolomics’ analyzes were performed by combining supernatants from two different solvent precipitation processes: (i) 400 µL of sodium-phosphate buffer (pH 7.0) was added to 400 µL of whole blood and mixed. Then, 1200 µL of acetonitrile was added, vortexed for 10 seconds, and centrifuged at 13,000 g. The exact process was repeated (ii) in another 400 µL of sodium-phosphate buffer (pH 7.0) previously added to 400 µL of whole blood and mixed, but replacing the acetonitrile by 1200 µL of methanol, vortexed for 10 seconds, and centrifuged at 13,000 g. Both supernatants were transferred to a new test tube and mixed. The organic solvents were evaporated at nitrogen flow, and the extracted resuspended in 1 mL of 2% acetic acid-water solution for the analysis by LC-HRMS.

#### LC-HRMS Analysis

The analytical platform used was an AccelaLC (ThermoFisher Scientific, Bremen, Germany) coupled to a QExactive Orbitrap Plus (MS) mass spectrometer (ThermoFisher Scientific, Bremen, Germany), as previously described (24, 25). The instrument was calibrated in positive and negative modes to guarantee mass accuracy below six ppm using the manufacturer’s calibration solutions (ThermoFisher Scientific, Bremen, Germany). Non-Target statistical analyzes were performed using a differential analysis platform, the SIEVE^®^ software version 2.1 (ThermoFisher Scientific, Rockford, USA). The masses selected from the analysis of SIEVE were searched for in two different databases of human metabolites: Human Metabolome Database (HMDB; The metabolomics Innovation Center, Canada, USA) and MetabolomicsDatabase (METLIN ™; California, USA).

#### Chemometrics

The raw data acquired with LC–HRMS were directly processed with Sieve applying the experiment type defined as “Small Molecule,” “Chromatographic Alignment and Framing,” and “Two Sample Differential Analysis” options, as previously described (26). Settings during Sieve operation were defined with a window of m/z 100–800; 2.5-minute frame time window; 6 ppm frame m/z width, and retention time of 0.01–11 minutes. The MS frame was set to all chromatographic peaks with a signal-to-noise threshold > 3, without the exclusion of isotopes. The metabolic composition of the data set for statistical evaluation consisted of all matrix components recovered by the analytical procedures as described by de Oliveira Sardela (26). Sieve^®^ software operated in two steps, comprising a first alignment of the chromatogram employing a ChromAlign™ algorithm and a second, a recursive base-peak framing, where spectral data from samples in the experiment were grouped by relative intensity. The data matrix was constructed by Sieve with the samples as observations, the m/z values, and retention time pairs as the response variables, identifying statistically significant abundance differences in the signal intensity of m/z values among different samples.

### Statistical Analysis

Data are expressed as means ± standard error of the mean (SEM). The comparisons of means to quantitative variables between groups were performed using Student’s t-test or ANOVA one-way test, followed by Bonferroni post-test. For all analyzes, a value of *p* < 0.05 was considered statistically significant. Statistical evaluation was performed using GraphPad Prism™ software version 7.0 (GraphPad Software, La Jolla, CA).

## Results

### Release of Heme and EVs During Cold Storage

**Figure 2:**
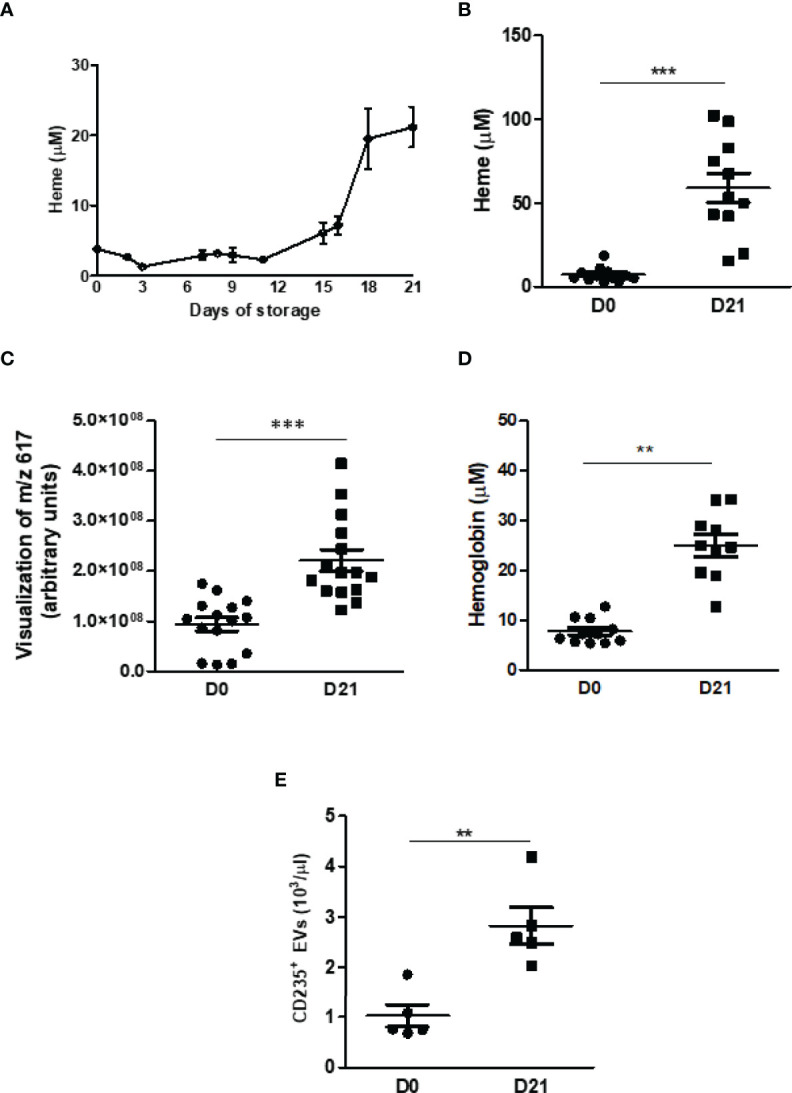
Quantification of heme, hemoglobin, and EVs in plasma. **(A)** Free heme concentrations were measured throughout 21 days. The analysis was performed *via* absorbance and read at 398 nm (n = 4). **(B)** Free heme concentrations were measured on day 21. The analysis was performed *via* absorbance and read at 398 nm (n = 11) and metabolomics and chemometric data evaluation *via* e the abundances of the ion m/z 617 **(C)**. **(D)** Hemoglobin concentrations were measured on day 21. The analysis was performed *via* absorbance and read at 540 nm (n = 11). **(E)** The MPs were isolated from the plasma of fresh blood samples or previously stored at 4°C for 21 days. After centrifugation (70 min, 20000 x g, 4°C), they were resuspended in a buffer for specific binding. MP positive for the glycophorin protein (CD235) were quantified and are expressed as MP/μl plasma (n=6). Results are expressed as mean ± standard error (SEM). **Represents p < 0.01, ***represents p < 0.005.

According to the literature, the storage of blood at 4°C leads to RBC injury even in the presence of blood stabilizers, with hemolysis and the release of RBC intracellular contents (27) and RBC derived EVs (28–30) into the bloodstream. Corroborating these data, the results presented herein demonstrated increased free heme levels starting from day 15, further augmenting daily until day 21 (**Figure 2A**). Analysis by absorption spectroscopy revealed an 8-fold increase of free heme released in plasma from stored RBC (**Figure 2B**), further corroborated by metabolomics and chemometric data evaluation, which has shown changes in the abundances of the ion m/z 617, identified as the heme (**Figure 2C**). Furthermore, hemoglobin plasma levels were 3.2-fold increased (**Figure 2D**), and RBC-EVs levels were 2.7-fold-increased (**Figure 2E**).

### Gene Expression Profile of Monocytes Co-Cultured With Cold-Stored RBC

**Figure 3:**
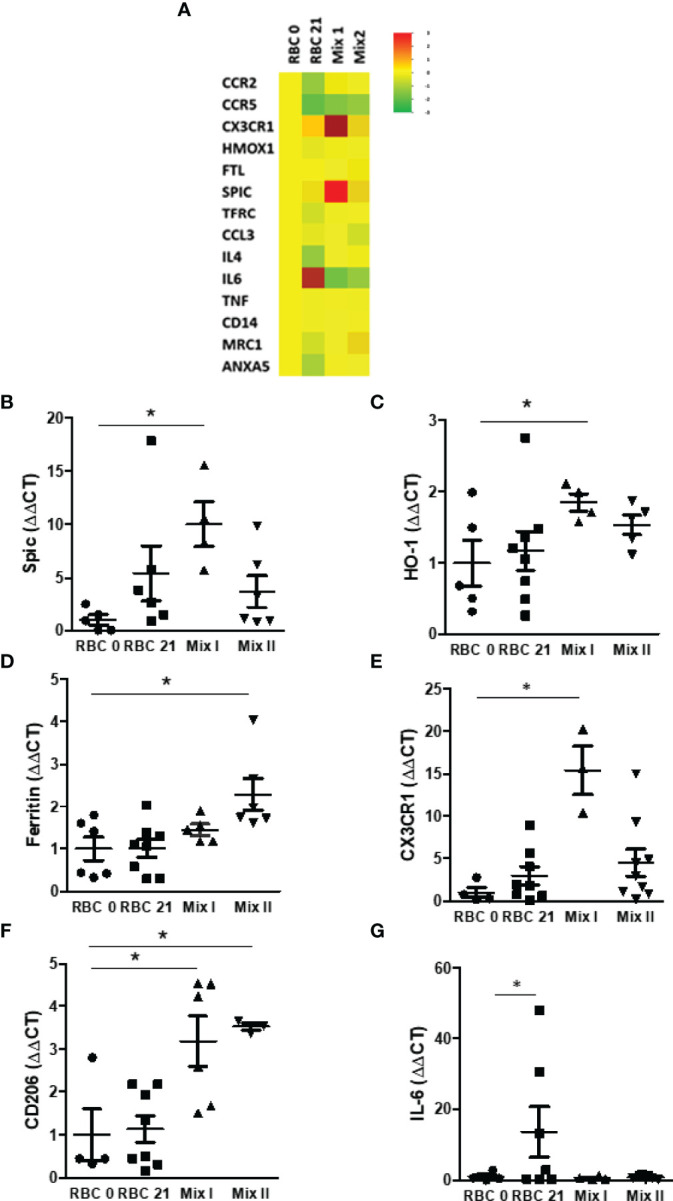
Comparison of genes differentially expressed between monocytes. **(A)** mRNA was purified from monocytes co-cultured in four different conditions: RBC 0: MØ + 100% fresh RBC; RBC 21: MØ + 100% stored RBC for 21 days at 4°; Mix 1: MØ + 80% fresh RBC + 20% stored RBC for 21 days at 4°C and Mix 2: MØ + 90% fresh RBC + 10% stored RBC for 21 days at 4°C. Data are expressed as mean relative gene expression in arbitrary units. **(B)** HO-1, **(C)** Spic, **(D)** ferritin, **(E)** CX3CR1, **(F)** CD206, and **(G)** IL-6 mRNA expression in monocytes was measured by TLDA in four different conditions: RBC 0: MØ + 100% fresh RBC; RBC 21: MØ + 100% stored RBC for 21 days at 4°; Mix 1: MØ + 80% fresh RBC + 20% stored RBC for 21 days at 4°C, and Mix 2: MØ + 90% fresh RBC + 10% stored RBC for 21 days at 4°C. Results are expressed as 3 - 6 different experiments. Data are expressed as mean ± standard error (SEM). *Represents p < 0.05.

It is already known that monocytes are sensitive to free heme (8) and EVs (31) undergoing functional reprogramming to maintain homeostasis. Therefore, we have decided to evaluate the effect of the RBC on the expression of 14 selected candidate genes in monocytes. The results have shown that RBC storage has changed genes’ expression related to heme metabolism, macrophage phenotype, and inflammatory mediators **Figure 3A**). The mRNA levels of Spic and HO-1 induced by Mix II were 10-fold and 1.8-fold increased, respectively (**Figures 3B, C**). However, ferritin mRNA levels increased when monocytes were co-cultured with Mix II (**Figure 3D**). Regarding monocyte/macrophage phenotype, CX3CR1 levels were 15-fold increased by Mix I (**Figure 3E**), and CD206 mRNA levels were 3-fold increased by Mix I and Mix II (**Figure 3F**). Mix I and Mix II represent moderate free heme levels (13 to 18 μM), while RBC21 represents very high levels (60 μM), inducing a 13-fold increase in IL-6 mRNA expression (**Figure 3G**), characteristic of proinflammatory response.

### Expression of Heme Metabolizing Proteins in Monocytes Co-Cultured With RBC

**Figure 4:**
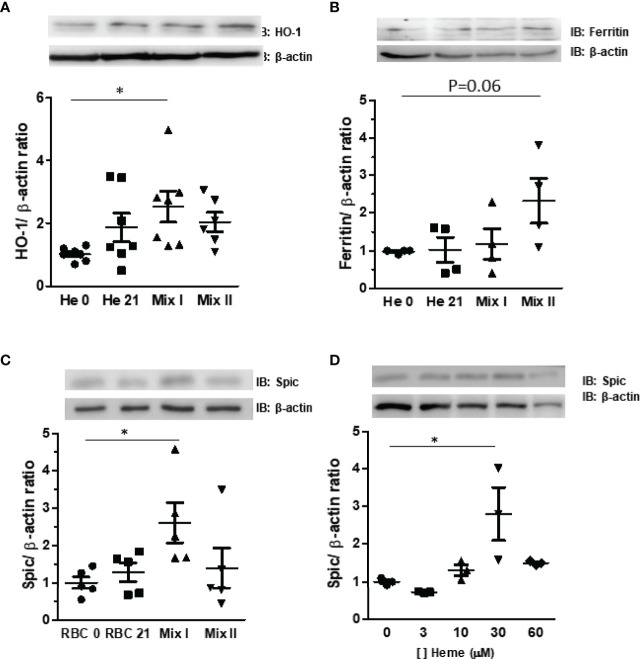
Effect of storage of RBC under protein expression in monocytes. Monocytes (MØ) were co-cultured with RBC for 24 hours in four different conditions: RBC 0: MØ + 100% fresh RBC; RBC 21: MØ + 100% stored RBC for 21 days at four °C; Mix I: MØ + 80% fresh RBC + 20% stored RBC for 21 days at 4°C, and Mix II: MØ + 90% fresh RBC + 10% stored RBC for 21 days at 4°C Protein extracts were subjected to Western Blotting for quantification of **(A)** HO-1, **(B)** ferritin, and **(C)** Spic expression. The results are representative of four to seven different experiments. **(D)** Monocytes (MØ) were incubated with heme at concentrations of 3, 10, 30, and 60 μm. The protein extracts were submitted to Western Blotting for quantification of Spic levels. The results are representative of three different experiments. Data are expressed as mean ± standard error (SEM). *Represents p < 0.05.

Our results have demonstrated that cold-stored RBCs undergo hemolysis which alters the monocytes’ mRNA expression of genes committed to heme metabolism. In this regard, we have further investigated the expression levels of heme metabolizing proteins. Corroborating data from the mRNA analysis, our results have demonstrated a 2.6-fold increase in HO-1 (**Figure 4A**) and a trend-to-increase in ferritin (**Figure 4B**) protein expression induced by Mix I and Mix II, respectively. Spic is known as a transcription factor that activates the differentiation of monocytes when in the presence of heme (8). We have observed that Mix II induced a 2.9-fold increase of Spic in monocytes while Mix II did not (**Figure 4C**). To further highlight cues regarding Spic expression, we have incubated monocytes with different heme concentrations. Our results have demonstrated that 30 μM of heme increased 2.8-fold Spic expression levels, while both 10 and 60 μM did not induce an increase in Spic protein expression (**Figure 4D**).

### Effect of Heme-Enriched EVs-RBC on Monocytes

**Figure 5:**
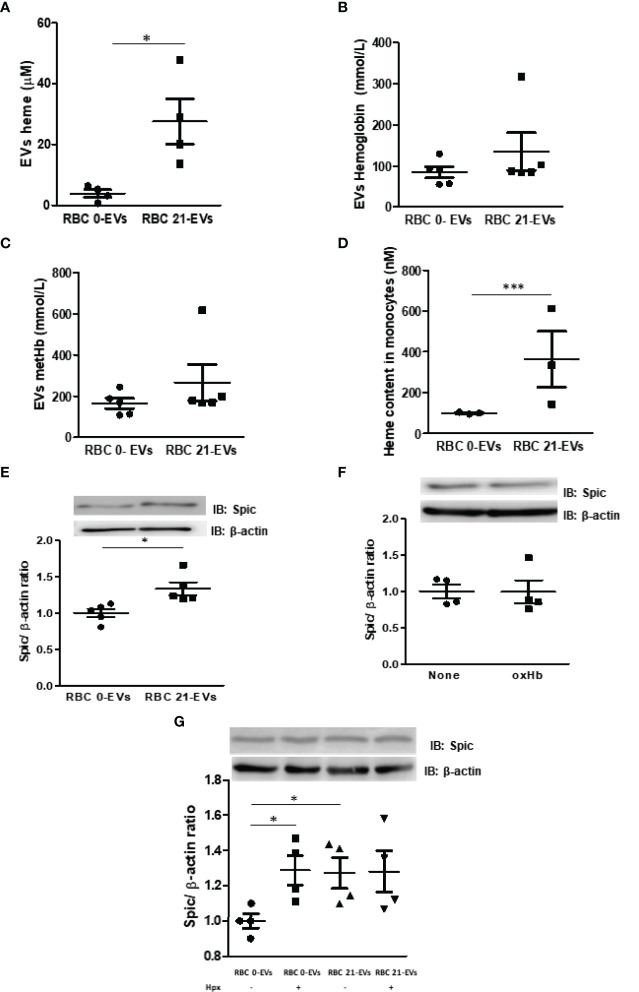
Characterization of RBC-EVs and their effects in monocytes. EVs were isolated from fresh and 21 day-stored-blood by centrifugation at 20,000 x g **(A)** Heme and **(B)** hemoglobin within EVs were measured using a plate reader at 398 nm and 540. **(C)** Oxidized hemoglobin (metHb) was measured at 541, 576 and 630 nm by the following equation: [metHb] = 185.77 x OD541+ 171.88 X OD576 +387.58 X OD630. **(D)** Isolated monocytes were challenged with 20% (v/v) EVs (fresh or stored) for 30 min, washed and lysed with RIPA buffer on ice, and captured heme was measured at 398 nm within monocytes. **(E)** Monocytes were treated for 24h with 20% (v/v) of fresh (RBC 0-EVs) or stored EVs (RBC 21-EVs), and protein extracts were subjected to Western Blotting for quantification of Spic expression. **(F)** Monocytes were treated for 24 hours with metHb at a concentration of 30 µM of heme, and protein extracts were subjected to Western Blotting to quantify Spic expression. **(G)** Monocytes were treated for 24 hours with RBC 0-EVs, and RBC 21-EVs previously incubated or not with hemopexin (Hpx; 1 µM for 1 hour), and protein extracts were subjected to Western Blotting for quantification of Spic expression. The results are representative of 3 to 5 different experiments. Data are expressed as mean ± standard error (SEM). *Represents p < 0.05; ***represents p < 0.005.

**Figure 6:**
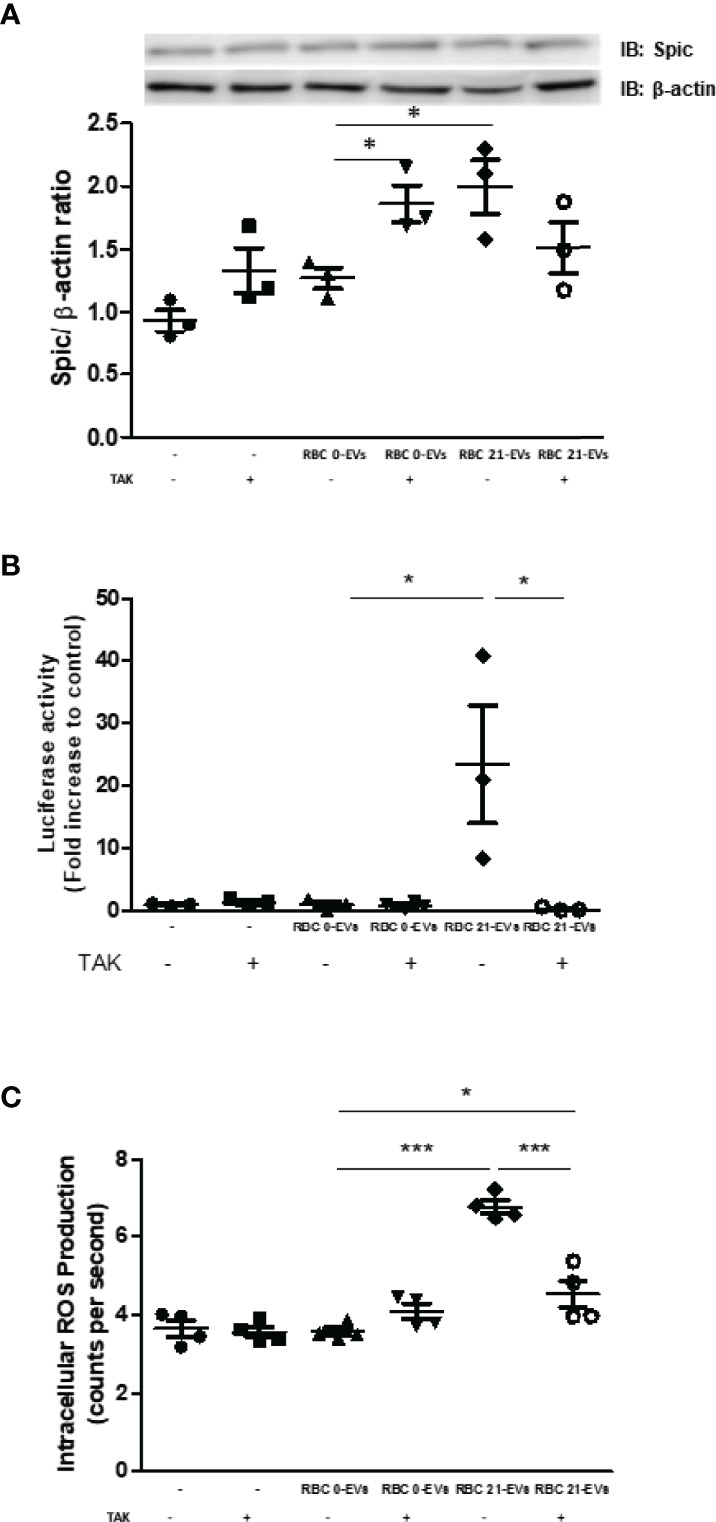
Role of TLR4 in RBC-EVs driven monocytes activation. **(A, B)** Monocytes were pretreated or not with TAK-242 (1 µM) or DMSO (0.001%) for 15 minutes and then incubated with RBC 0-EVs or RBC 21-EVs for 24 hours. After that, **(A)** protein extracts were subjected to Western Blotting for quantification of Spic expression, and **(B)** NF-kB activation was evaluated by luciferase activity. **(C)** ROS production was assessed by monocytes uptake of the CellROX probe for 30 min. After that time, cells were washed and preincubated with TAK-242 (1µM) or DMSO (0.001%) for 15 minutes at 37°C. After the pretreatment, cells were treated with RBC 0-EVs, RBC 21-EVs, for 15 minutes and had the excitation and emission of the fluorescence monitored, respectively, at 640 and 665 nm. The results are representative of 3 -4 different experiments. Data are expressed as mean ± standard error (SEM). * Represents p < 0.05 and *** represents p < 0.005.

EVs carry the cargo of mother cells, including DNA, messenger mRNA, miRNAs, proteins, lipids, and metabolites, to acceptor cells (18, 31). It is known that heme is found in EVs-RBC in SCD patients (18). Herein, we aimed to unravel if long-term cold storage can increase heme and hemoglobin released by RBC within EVs. We have observed a 7.2-fold increased heme content within EVs released by cold-stored RBC (RBC 21-EVs) compared to EVs released by fresh RBC (RBC 0-EVs) (**Figure 5A**). Conversely, both RBC 0-EVs and RBC 21-EVs presented the same content of hemoglobin (**Figure 5B**) and metHb (**Figure 5C**). Next, we have investigated if RBC-EVs target and activate monocytes. After incubating monocytes with RBC-EVs, and washing, heme was quantified. Our results demonstrated that RBC 21-EVs transferred a 3.6-fold increased heme content to monocytes in comparison to RBC 0-EVs (**Figure 5D**). Finally, it was evaluated if the heme transfer from cold-stored RBC to monocytes *via* EVs could impact in protein expression of Spic, and interestingly our results have revealed a more significant amount of Spic in monocytes previously incubated with RBC 21-EVs than with RBC 0-EVs (p<0.05) (**Figure 5E**). We have further evaluated if metHb – a known RBC-derived DAMP- could play a role in RBC-EVs-driven monocyte activation. Our results demonstrated no differences in Spic content between monocytes left untreated or treated with oxidized hemoglobin (**Figure 5F**). Hemopexin (Hpx) is known to scavenge cell-free heme providing systemic protection against its deleterious effects (32). Thus, we aimed to evaluate if the pretreatment of RBC-EVs could impact Spic expression in monocytes. Our results demonstrated that Hpx had no impact under Spic expression triggered by stored-RBC EVs in monocytes (**Figure 5G**). It is known that heme can trigger TLR4 signaling pathways in immune cells (33). Thus, to understand the mechanisms underlying EVs-driven Spic expression in monocytes, we evaluated the role of TLR4 signaling pathways in this scenario. Our results have demonstrated that the pretreatment of monocytes with TAK-242 (TLR4 antagonist) did not prevent the upregulation of Spic by RBC 21-EVs (**Figure 6A**). Furthermore, we have observed that RBC 21-EVs increased NF-kB activity in monocytes in a TLR4 dependent manner once TAK-242 prevented this effect (**Figure 6B**). Our results have also demonstrated that RBC 21-EVs induced ROS in monocytes at 15 minutes (p<0.005), which was partially prevented by TAK-242, thus suggesting that RBCs 21-EVs induce ROS both dependently and independently of TLR4 (**Figure 6C**).

## Discussion

In the present work, an *ex vivo* protocol was adopted by co-cultivating monocytes with 100% fresh, 100% refrigerated, or mixed proportions of RBC (20% stored + 80% fresh; 10% stored + 90% fresh) to mimic autologous blood transfusion. Initially, we observed that hemolysis induced by 21-day-cold storage induced the release of large amounts of free heme, hemoglobin, and EVs in plasma. Therefore, we investigated whether such metabolites could impact the expression of genes related to heme metabolism and monocytes profile. Notably, the amount of free heme has impacted the monocytes’ phenotype differently. For example, low amounts of heme led to increased expression of molecules related to heme metabolism, such as Spic, HO-1, and ferritin, along with molecular markers that characterize anti-inflammatory monocytes/macrophages (M2 polarization) such as CX3CR1 and CD206. Furthermore, very high amounts of heme induced the expression of IL-6, a classic proinflammatory (M1 polarization) mediator. One may highlight the increased expression of Spic, as it is classically known as a transcription factor present in red pulp macrophages. To understand how communication between red blood cells and monocytes would be supported, we evaluated the role of EVs in this context. We have further observed that EVs released by RBC during cold storage contained elevated free heme concentrations, which were transferred to monocytes, inducing the production of ROS and Spic.

The key finding of our study was to analyze the effects of hemolysis caused by RBC storage on monocytes. To the best of our knowledge, this strategy of looking to monocytes seeking molecular signatures triggered by blood transfusion has never been explored. So, we aimed to study the changes undergone in monocytes after contact with previously-stored RBC. The idea presented herein is conceivable with previous results from Pottgiesser et al., demonstrating that reinfused stored RBC triggers immune reactions within T-lymphocytes. According to them, ABT increases transcript levels of genes coding proteins that regulate T-lymphocyte activation, adaptive immune response, Toll-Like-Receptor (TLR) pathways, cell apoptosis, and endocytosis surface receptors (34). D’Alessandro et al. conducted an elegant study analyzing the plasma metabolome of 18 healthy volunteers before and after reinfusion of 42 day-stored RBC, showing that metabolic changes undergone by stored RBC increased the levels of pro-oxidative, proinflammatory, and immunomodulatory metabolites in the plasma of healthy individuals (35).

It is consensual that several changes occur to stored RBC, triggering an enhanced release and accumulation of RBC-derived EVs over storage time (36). These EVs have immunomodulatory effects (37), can be identified by the presence of glycophorin A (38), and are considered one of the expression markers of storage lesions (39).

The present study hypothesized that the increased release of free heme by previously-stored RBCs could trigger monocyte immune responses. Our study’s main finding is that stored RBC induces a distinct phenotype in transcriptomic and protein profiles in monocytes. The transcriptomic analysis revealed that when co-cultured with 20% of stored RBC, monocytes underwent molecular changes towards an anti-inflammatory M2-like phenotype. Such findings corroborate previous reports for macrophages exposed to heme (40).

More precisely, our results showed increased CX3CR1 and CD206 mRNA levels, both known as M2 macrophage markers (41). CD206, for example, is induced in macrophages stimulated with hemin. Peripheral blood monocytes from hemolytic patients, which undergo heme toxicity, preferentially differentiate towards a proinflammatory M1-like phenotype (42), suggesting that even though heme is known to induce an M2-like phenotype, very high levels of heme can induce a proinflammatory response. Intriguingly, when monocytes were incubated with 100% of stored RBC, both HO-1 mRNA and protein expression and CX3CR1 and CD206 mRNA levels were not significantly increased, and these monocytes displayed these increased proinflammatory IL-6 mRNA levels, as previously reported (43).

It has already been demonstrated that NF-κB/p65 antagonizes Nrf2-ARE dependent pathways, and since HO-1 expression is driven by Nfr2 (44, 45), it could explain why the HO-1 expression levels in the RBC 21 group were not increased. Gbotsho et al. recently demonstrated that when the plasma heme scavenging system is saturated, the circulating heme induces a cardiac expression of IL-6 in sickle cell mice through an Nfr2 independent pathway. Even though the underlying mechanisms of heme-induced IL-6 expression still need to be addressed, the authors suggested that this mechanism might involve the TLR-4-MyD88-AP-1 NF-ĸB axis (46).

High levels of circulating free heme induce an inflammatory response in mononuclear cells compared with an inflammatory response triggered by endotoxin (47). Small concentrations of heme act as an anti-inflammatory and cytoprotective agent *via* upregulating HO-1 and stimulating the formation of HO-1 end products, such as CO and biliverdin (48, 49), whereas a large amount of heme is deleterious on tissues *via* its pro-oxidative and proinflammatory function (50). Monocytes correlate positively with hemolysis markers, and their absolute number is increased in hemolytic anemias, reinforcing the impact of heme and hemolysis on monocyte/macrophage development (51).

Camus et al. have demonstrated that a proportion of cell-free heme is within RBC-EVs, and, when incubated with endothelial cells or infused into SCD mice, can mediate endothelial injury. In this seminal paper, they have proposed that EVs convey heme directly from its accumulation in membranes before vesiculation or from cytoplasm transfer during vesiculation. A plausible explanation is due to heme’s highly lipophilic and membranotropic characteristics (18). The EVs accumulation triggered by the accelerated RBC aging process during storage has emerged as an essential focus of study in transfusion medicine. It has been shown that EVs can mediate immunomodulatory effects (37). For example, EVs from older stored blood bags accumulated prime neutrophils and triggered inflammatory responses in transfused patients (52). Although the exact mechanisms of transfusion-related immunomodulation are not still understood, possible the mechanisms include suppression of cytotoxic cells and monocyte/macrophages activity (37). It was already demonstrated that hemolysis alters actin cytoskeleton remodeling, triggering decreased phagocytic capacity (53). Corroborating, our findings have shown that stored RBC-EVs presented high amounts of heme, which was transferred to monocytes, triggering acute ROS production dependently and independently of TLR4, suggesting a dual role of RBC-EVs in monocytes activation. In agreement, it has already been demonstrated that heme-enriched EVs convey heme to endothelial cells, triggering oxidative stress, which is nearly entirely prevented by TLR4 neutralization (18). It is worth mentioning that free heme (30 µM) induces ROS in peritoneal murine macrophages in a TLR4 independent manner (33). One of the main findings of our study was to demonstrate heme-enriched RBC-EVs released from stored RBC (20% v/v) increased the expression of Spic in monocytes. The present study us the first to study showing that monocytes increase Spic expression in transfusion context to the best of our knowledge. Also, we have shown that both mRNA and protein expression of Spic is increased in monocytes co-cultured with stored RBC. Apart from its role in iron-recycling macrophages development, Spic has emerged as a transcription factor involved in inflammatory responses in monocytes and macrophages. It was previously reported that prolonged fasting increases Spic in human macrophages. Authors have demonstrated that exposure to fatty acids induces Spic in circulating monocytes, suggesting Spic as a regulator of mechanisms interfering in macrophages differentiation (11). Our findings have also shown that stored RBC increases classic M2-like macrophage markers, such as CD206, HO-1, CX3CR1, and Spic. Accordingly, Alam et al. demonstrated Spic mediated downregulation of proinflammatory cytokines in macrophages (10), and Kayama et al. showed the downregulation of inflammation in intestinal macrophages in a Spic dependent manner (54).

**Figure 7:**
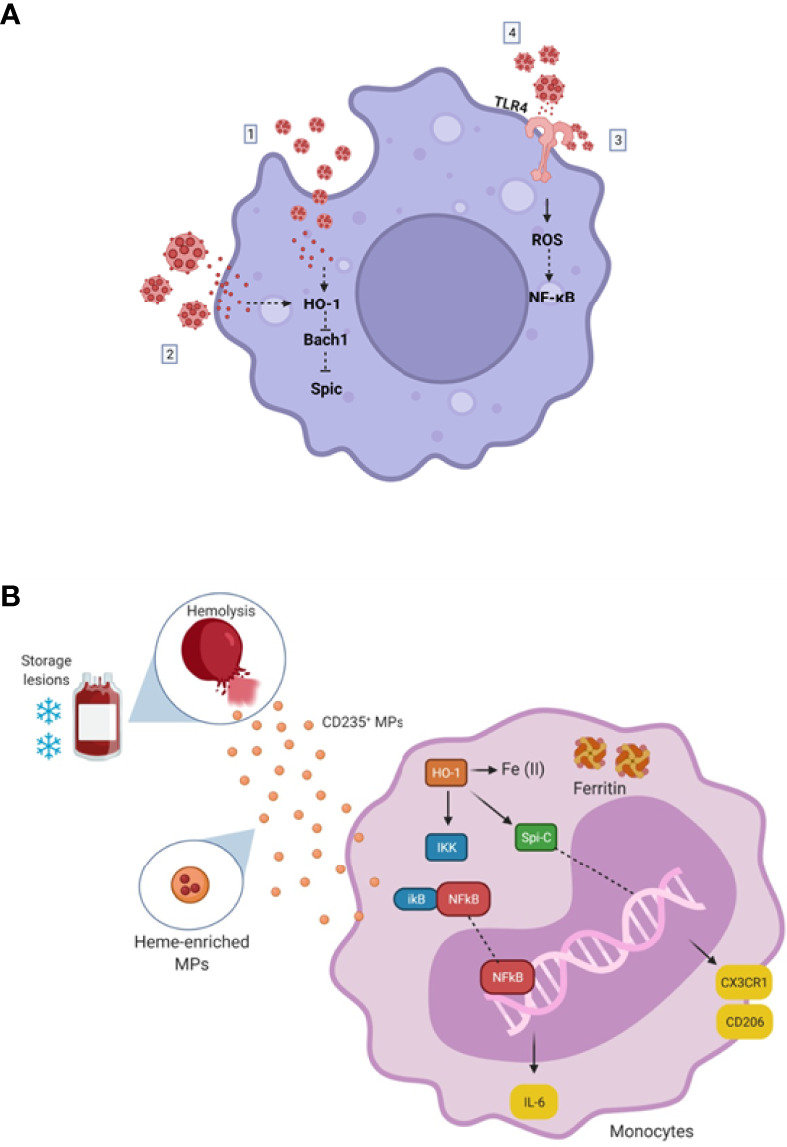
**(A)** Proposed model of RBC-EVs driven monocytes activation. (1) RBC-EVs enriched in heme are endocytosed by macrophages, triggering HO-1 expression, possibly culminating in Bach1 repression leading to Spic expression. (2) RBC-EVs release heme content near the plasma membrane of macrophages; free-heme crosses the membrane triggering HO-1 expression, probably culminating in Bach1 repression leading to Spic expression. (3) Heme contained in RBC-EVs membrane activates TLR4 directly, triggering intracellular ROS production, probably activating NF-kB. (4) RBC-EVs release heme content near the plasma membrane of macrophages; free-heme activates TLR4, triggering intracellular ROS production, activating NF-kB. **(B)** Simplified representative scheme for changes caused by blood storage. Blood storage leads to red cell insults, causing hemolysis and releasing heme, hemoglobin, and MPs into the bloodstream. Heme activates the expression of the Spic transcription factor, which signals the differentiation of monocytes/macrophages into an M2-like phenotype, expressing increased CD206 and CX3CR1 mRNA levels. Heme is degraded by HO-1 generating Fe (II), CO, and Biliverdin in the monocytes. Fe (II) is captured by ferritin, avoiding oxidations. Additionally, when in very high concentrations, heme seems to activate the NF-κB proinflammatory signaling pathway, increasing IL-6 mRNA levels. (This scheme does not represent the dimensions and sizes in your reality). Created with BioRender.com.

We propose that Spic increase driven by RBC 21-EVs in monocytes is heme dependent since the content of Hb within EVs was not affected by cold storage. Corroborating this finding, the treatment of monocytes with oxidized hemoglobin did not increase Spic expression. Considering that Hpx, a heme scavenger, did not alter Spic expression in monocytes driven by RBC 21-EVs, we suggest that the heme contained in the interior of EVs triggered Spic expression in monocytes (**Figure 7A**). Alam et al. demonstrated that TLR activation induces Spic by an NF-kB dependent and heme-independent mechanism (10). Accordingly, our results have demonstrated that the inhibition of TLR4 did not affect Spic induction by RBC 21-EVs. Thus, we propose that RBC-EVs induce Spic through heme-mediated degradation of Bach1 (**Figure 7A**), as previously demonstrated by Haldar et al. (8).

Donati et al. simulated an *ex vivo* ABT protocol and demonstrated an 8-fold increase in EVs in blood samples stored for 30 days, concluding that EVs (MPs/μL) concentration could be a promising marker of blood transfusion (55). The US Food and drug administration (FDA) guidelines describe that only 75% of erythrocytes are recoverable at the end of the storage period, suggesting that further hemolysis and EVs formation occur *in vivo* after transfusion (56). However, studies concerning the clearance of these EVs are still needed to clarify how long these EVs can be detectable after ABT.

In conclusion, hemolysis resulting from ABT alters mRNA and proteins expression in monocytes towards an M2 phenotype (**Figure 7B**). One may highlight the role of EVs released by 21-day-stored RBC as an inductor of Spic in this scenario. Together, our results help increase the knowledge of immunomodulation triggered by blood storage and may open new anti-doping strategies where monocytes can be faced as an ABT sensor.

## Data Availability Statement

The raw data of LC-HMRS presented in this study can be found online at be found in online at figshare repository: https://doi.org/10.6084/m9.figshare.19161155.v1.

## Ethics Statement

The studies involving human participants were reviewed and approved by Universidade do Estado do Rio de Janeiro. CAAE 36880914.0.0000.5259. The patients/participants provided their written informed consent to participate in this study.

## Author Contributions

Conceptualization, MR-M and JM. Writing—original draft preparation, CA and MR-M. Writing—review and editing, FA, HP, and MR-M. Visualization, LM, AC, JM, and MR-M. Conduction of experiments, CA, SL, LM, AC, JM, IN, VS, LP, IM, and MR-M. Supervision, HP and MR-M. All authors contributed to the article and approved the submitted version.

## Funding

This work was supported by a grant from the Partnership for cleaning competition and the Brazilian Doping Control Authority.

## Conflict of Interest

The authors declare that the research was conducted in the absence of any commercial or financial relationships that could be construed as a potential conflict of interest.

## Publisher’s Note

All claims expressed in this article are solely those of the authors and do not necessarily represent those of their affiliated organizations, or those of the publisher, the editors and the reviewers. Any product that may be evaluated in this article, or claim that may be made by its manufacturer, is not guaranteed or endorsed by the publisher.
